# Purines released from astrocytes inhibit excitatory synaptic transmission in the ventral horn of the spinal cord

**DOI:** 10.3389/fncir.2014.00060

**Published:** 2014-06-04

**Authors:** Eva Meier Carlsen, Jean-François Perrier

**Affiliations:** Laboratory of Neuronal Signaling, Department of Neuroscience and Pharmacology, University of CopenhagenCopenhagen, Denmark

**Keywords:** astrocyte, ATP, adenosine, purine, spinal cord, synapse, motor control

## Abstract

Spinal neuronal networks are essential for motor function. They are involved in the integration of sensory inputs and the generation of rhythmic motor outputs. They continuously adapt their activity to the internal state of the organism and to the environment. This plasticity can be provided by different neuromodulators. These substances are usually thought of being released by dedicated neurons. However, in other networks from the central nervous system synaptic transmission is also modulated by transmitters released from astrocytes. The star-shaped glial cell responds to neurotransmitters by releasing gliotransmitters, which in turn modulate synaptic transmission. Here we investigated if astrocytes present in the ventral horn of the spinal cord modulate synaptic transmission. We evoked synaptic inputs in ventral horn neurons recorded in a slice preparation from the spinal cord of neonatal mice. Neurons responded to electrical stimulation by monosynaptic EPSCs (excitatory monosynaptic postsynaptic currents). We used mice expressing the enhanced green fluorescent protein under the promoter of the glial fibrillary acidic protein to identify astrocytes. Chelating calcium with BAPTA in a single neighboring astrocyte increased the amplitude of synaptic currents. In contrast, when we selectively stimulated astrocytes by activating PAR-1 receptors with the peptide TFLLR, the amplitude of EPSCs evoked by a paired stimulation protocol was reduced. The paired-pulse ratio was increased, suggesting an inhibition occurring at the presynaptic side of synapses. In the presence of blockers for extracellular ectonucleotidases, TFLLR did not induce presynaptic inhibition. Puffing adenosine reproduced the effect of TFLLR and blocking adenosine A_1_ receptors with 8-Cyclopentyl-1,3-dipropylxanthine prevented it. Altogether our results show that ventral horn astrocytes are responsible for a tonic and a phasic inhibition of excitatory synaptic transmission by releasing ATP, which gets converted into adenosine that binds to inhibitory presynaptic A_1_ receptors.

## INTRODUCTION

Neuronal networks located in the ventral horns of the spinal cord are essential for motor control (Kjaerulff and Kiehn, 1996; Prut and Perlmutter, 2003; Lanuza et al., 2004; Dai et al., 2005; Brocard et al., 2010; Mui et al., 2012). Their architecture provides the necessary stability for generating stereotyped movements. In order to adapt to internal and external changes, the organism adjusts neuronal activity by different cellular mechanisms providing high degrees of flexibility on time scales ranging from milliseconds to hours. The activity of spinal networks is regulated by descending pathways releasing neuromodulators such as serotonin, noradrenaline, glutamate or GABA (Arvidsson et al., 1990; Jacobs and Azmitia, 1992; Ono and Fukuda, 1995; Schmidt and Jordan, 2000; Conn et al., 2005; Farrant and Nusser, 2005; Perrier and Delgado-Lezama, 2005; Cotel et al., 2013; Perrier et al., 2013). Flexibility is also ensured by intrinsic modulatory systems releasing transmitters such as endocannabinoids (Ameri, 1999; El Manira and Kyriakatos, 2010) or purines (Dale and Gilday, 1996). The modulation provided by purines is of particular interest because adenosine triphosphate (ATP) and its metabolic product adenosine exert opposite effects on neurons. In the spinal cord of embryonic *Xenopus*, ATP promotes swimming by inhibiting voltage gated potassium conductances (Dale and Gilday, 1996; Brown and Dale, 2002). Similarly, in the brainstem, ATP activates neurons from the retrotrapezoid nucleus, involved in breathing (Gourine et al., 2010). Once in the extracellular space, ATP is converted by ectonucleotidases into adenosine diphosphate (ADP), adenosine monophosphate (AMP), and adenosine (Dunwiddie et al., 1997). The binding of adenosine to metabotropic A_1_ receptors lowers the excitability of spinal neurons belonging to motor circuits by inhibiting Ca^2+^ currents (Dale and Gilday, 1996; Brown and Dale, 2000). In agreement, (Taccola et al., 2012) and (Brockhaus and Ballanyi, 2000) showed that A_1_ receptor activation depresses bicuculline-evoked seizure-like bursting in newborn rat spinal cords. In other systems such as the hippocampus or the cerebellum, the activation of presynaptic A_1_ receptors decreases neurotransmitter release by inhibiting Ca^2+^ channels (Hollins and Stone, 1980; Wu and Saggau, 1994; Zhang et al., 2003).

Sources of ATP in the nervous system are multiple. ATP is co-released from neurons with neurotransmitters such as acetylcholine, noradrenaline, or GABA (Silinsky and Redman, 1996; Jo and Schlichter, 1999; Burnstock, 2007). In addition, ATP is secreted from astrocytes (Guthrie et al., 1999; Fields and Burnstock, 2006; Gourine et al., 2010; Christensen et al., 2013; Lalo et al., 2014). In the spinal cord, some of the modulatory actions induced by ATP are blocked by the glial metabolic poison fluoroacetate (Witts et al., 2012). It was therefore suggested that spinal glial cells also release ATP. However, a direct demonstration that ATP released from spinal astrocytes modulates synaptic transmission is still lacking.

In this study, we investigated if purines released from spinal astrocytes have any effect on synaptic transmission between neurons from the ventral horn. We found that astrocytes release ATP, which after being converted to adenosine produces both tonic and phasic inhibition of excitatory synaptic transmission by decreasing the probability of neurotransmitter release.

## MATERIALS AND METHODS

**FIGURE 1:**
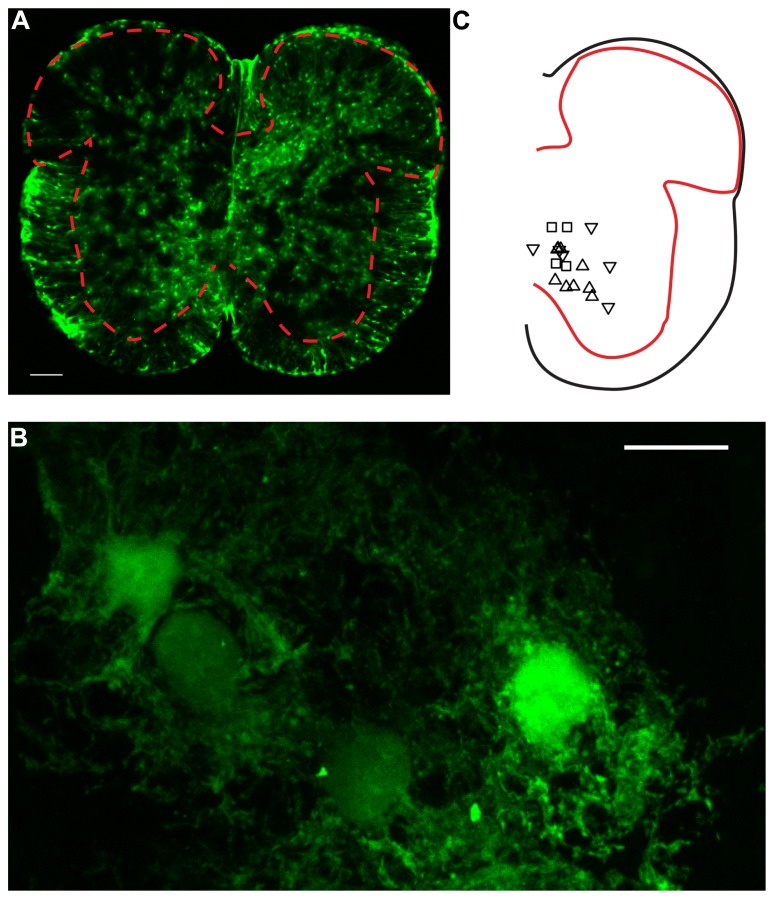
**Confocal microscopy of GFAP-EGFP astrocytes in spinal cord slices. (A)** Image of a lumbar spinal cord slice from a neonatal mouse (P7) scanned with confocal microscopy. The red dashed line indicates the limits between gray and white matter. Note the presence of multiple protoplasmic fluorescent astrocytes in the gray matter. Scale bar: 100 μm. **(B)** High magnification picture (×63) of ventral horn E-GFP positive astrocytes. Twenty-four consecutives parallel confocal plans were superimposed. Scale bar: 10 μm. **(C)** Relative positions of a fraction of the neurons recorded for the study. Upward triangles indicate responses paired pulse stimulation protocol with a PPR > 1. Downward triangles correspond to a PPR < 1. Squares: PPR≈1.

Experiments were performed on neonatal (P4–P22) wild type (C7BJL6; Taconic) and transgenic mice expressing enhanced green fluorescent protein (E-GFP) under the promoter of glial fibrillary acidic protein [GFAP; line Tgn(hgFAPEGEP)] GFEC 335 (**Figures 1A,B**). Transgenic mice were kindly provided by Professor Frank Kirchhoff. The astrocytes of these mice were identified by their fluorescence (Nolte et al., 2001). The surgical procedures complied with Danish legislation. Mice were killed by decapitation.

### SLICE PREPARATION

The lumbar enlargement of the spinal cord was removed and placed in cold artificial cerebrospinal fluid containing NaCl 125 mM, KCl 2.5 mM, NaHCO_3_ 26 mM, CaCl_2_ 2 mM, MgCl_2_ 1 mM, NaH_2_PO_4_ 1.25 mM, Glucose 25 mM. Ringer’s solution was continuously carbogenated by gassing with 95% O_2_ plus 5% CO_2_. 300 μm thick slices were obtained with a vibratome (MicroM slicer HM 650V; Microm International GmbH, Germany) equipped with cooling unit CU65 set at 2°C. Slices were then positioned in a recording chamber and continuously perfused with Ringer’s solution at room temperature.

### PATCH CLAMP RECORDING

Visual guided patch clamp recording was done with a Multiclamp 700B amplifier (Molecular Devices, USA). Neurons were visualized by means of a BW51WI microscope (Olympus, Japan) equipped with an oblique illumination condenser. Astrocytes were identified by means of fluorescence illumination obtained with a monochromator (Polychrome V; Till Photonics, Germany) tuned at 488 nm. The pipette solution (in mM): 122 K-gluconate, 2.5 MgCl_2_, 0.0003 CaCl_2_, 5.6 Mg-gluconate, 5 K-HEPES, 5 H-HEPES, 5 Na_2_ATP, 1 EGTA, 2.5 biocytine, 0.01 Alexa 488 hydrazide, sodium salt (Life Technologies, USA), and KOH to adjust the pH to 7.4. Calcium-clamp pipette solution contained (in mM): 40 K-gluconate, 30 K_4_-BAPTA, 50 sucrose, 2.5 MgCl_2_, 0.0003 CaCl_2_, 5.6 Mg-gluconate, 5 K-HEPES, 5 H-HEPES, 5 Na_2_ATP, 1 EGTA, 5 biocytine, 0.068 Alexa 568 hydrazide sodium salt (Life Technologies, USA) and the necessary amount of KOH to adjust the pH to 7.4. Electrodes had a resistance ranging from 4 to 8 MΩ. Recordings were sampled at 10 kHz with a 16-bit analog-to-digital converter (DIGIDATA 1440; Molecular Devices, USA) and displayed by means of Clampex 10.2 software (Molecular Devices, USA).

### ELECTRICAL STIMULATION

Local electric stimulation was performed with a bipolar concentric electrode (TM33CCNON; World Precision Instruments, Sarasota, FL, USA) connected to an isolation unit (Isolator 11, Axon Instruments, USA) triggered by an external signal. The electrode was positioned in areas devoid of cell bodies, in the vicinity of neurons recorded from. Different positions were tried until a reliable response was induced by stimulation. Responses to paired pulse stimulations (duration from 10 to 40 μs; amplitude from 0.1 to 1 mA; interval from 10 to 30 ms) were recorded in neurons in voltage-clamp mode. They were considered as putative excitatory monosynaptic postsynaptic currents (EPSCs) when all the following criteria were satisfied: (1) fixed latency and variable amplitude; (2) decrease of amplitude upon depolarization, as one would expect for EPSCs in contrast with IPSCs; (3) absence of response when reverting the polarity of stimulation to exclude possible artifacts caused by direct electrical stimulation. For a fraction of neurons, we also tested responses to stimulation at high frequencies (10, 20, and 50 Hz). The neurons that did not follow these frequencies were discarded from the sample.

### INPUT RESISTANCE

The input resistance of the neurons recorded from was calculated as the ratio of voltage to current measured during small depolarizing pulses generated in voltage-clamp mode.

### FOCAL APPLICATION OF DRUGS

Electrodes made from borosilicate capillaries (tip diameter ranging from 1.5–2 μm; BF150-86-7.5, Sutter Instrument, USA) were either filled with TFLLR-NH_2_ (10 μM; Tocris Bioscience, UK), ATP (1 mM; Sigma–Aldrich, St. Louis, MO, USA) or adenosine (1 mM; Sigma–Aldrich, St. Louis, MO, USA). Drugs were puff applied at 14–35 Pa by a homemade time-controlled pressure device.

### DRUGS

The following drugs were used: BAPTA (30 mM; Invitrogen, Carlsbad, CA, USA), 6-*N*, *N*-Diethyl-D-β,γ-dibromomethylene ATP trisodium salt (ARL 67156 trisodium salt, 50 μM; Tocris Bioscience, UK), 8-Cyclopentyl-1,3-dipropylxanthine (DPCPX, 5 μM; Tocris Bioscience, UK).

### CONFOCAL MICROSCOPY

Confocal microscopy images were obtained at the core facility for integrated microscopy (CFIM) of the Faculty of Health and Medical Sciences of the University of Copenhagen. Pictures were taken with a LSM 700 confocal microscope (Zeiss, Germany) equipped with Plan-Neofluar, X5 (N.A. 0.15) and Plan-Apochromat X63 (N.A. 1.4) objectives. E-GFP positive cells were excited with a 488 nm diode laser (10 mW).

### DATA ANALYSIS

Statistical analyses were performed offline by means of Origin software (version 8.6; MicroCal Inc., USA). The normality distribution of each sample was tested with Shapiro–Wilk test. Non-parametric tests were used for small samples (*n* < 10) and when Gaussian distribution could not be approximated. Data are represented as mean ± standard deviation of the mean.

## RESULTS

### ASTROCYTES PRODUCE TONIC PRESYNAPTIC INHIBITION IN THE VENTRAL HORN

**FIGURE 2:**
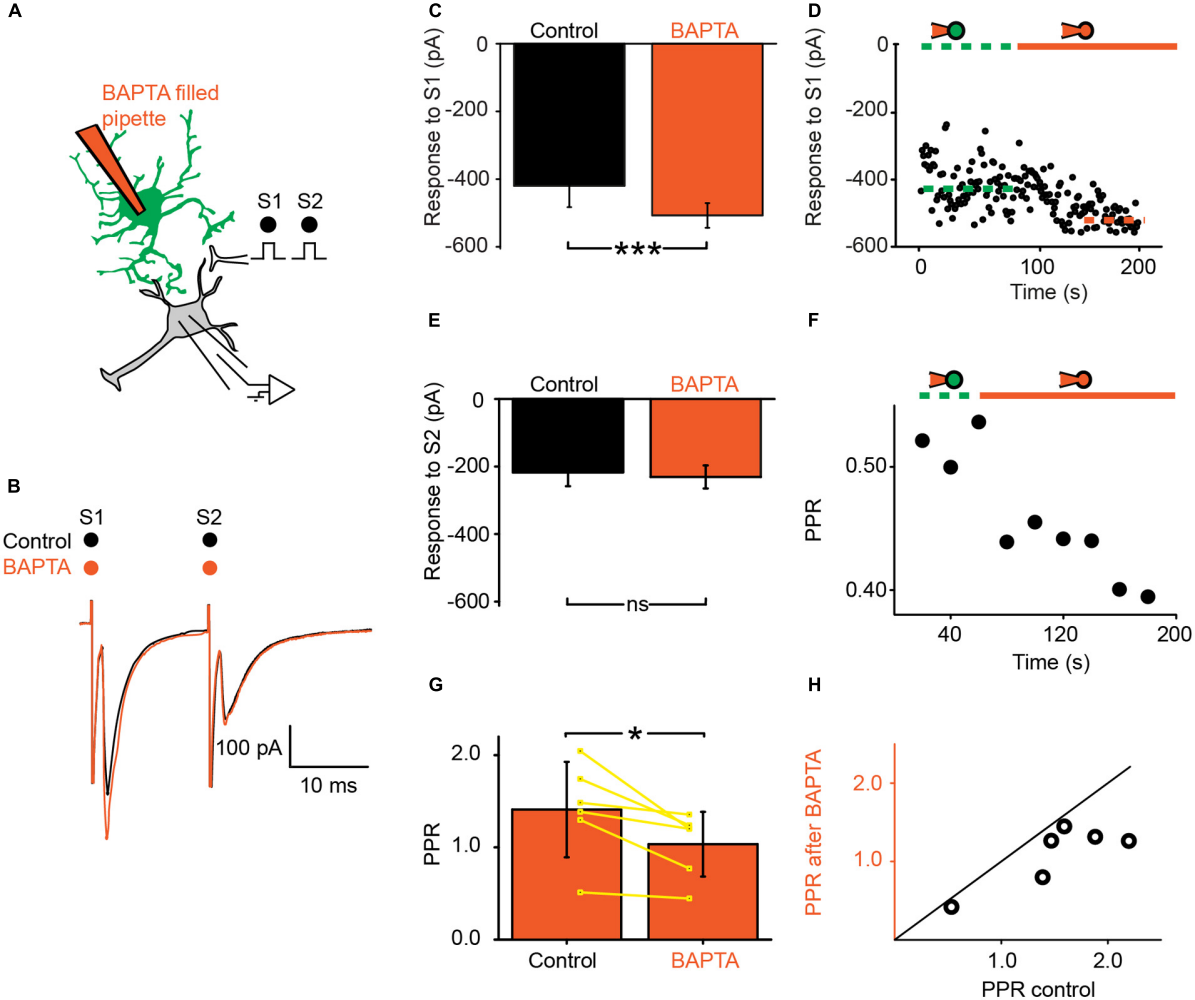
**Ventral horn astrocytes produce a tonic presynaptic inhibition of synaptic transmission. (A)** Schema of the principle of the preparation. Ventral horn neuron recorded in whole-cell mode. Synaptic stimulation produced by a bipolar electrode (S1, S2). Whole-cell recording of an astrocyte with 30 mM BAPTA. **(B)** Response of a ventral horn neuron to two stimulations in control conditions (black) and after chelating Ca^2+^ in one astrocyte (red). The amplitude of the first EPSC was increased. **(C)** Mean amplitude of the first EPSC in control condition and after chelating Ca^2+^ in one astrocyte. The amplitude was significantly increased. ^***^p < 0.001. **(D)** Amplitude of the first EPSC when the astrocyte was recorded in cell-attached configuration (doted green line) and after breaking-in in whole-cell configuration (red line). Note the gradual increase in amplitude. The dot lines correspond to the mean amplitudes in both conditions. **(E)** Amplitude of the second EPSC in control condition and after chelating Ca^2+^ in one astrocyte. **(F)** Time course of the changes occurring for the paired pulse ratio (PPR) calculated as the amplitude of the second response divided by the first for the neuron recorded in (B). Recording the astrocyte in whole-cell mode (red line) decreased the PPR. **(G)** Mean PPR for all the cells recorded before and after chelating Ca^2+^ in one astrocyte. The yellow lines correspond to individual pairs of values. The PPR was significantly decreased. ^*^p < 0.05. **(H)** PPR after Ca^2+^ chelation in one astrocyte as a function of PPR in control conditions. If BAPTA had no effect, the points would be distributed around the line of equality (black). All the points are below the line of equality.

We recorded ventral horn neurons (**Figure 1C**) from the spinal cord of neonatal mice with the whole-cell patch-clamp technique in voltage-clamp mode. Most of the neurons recorded from had a high input resistance (>500 MΩ) and were located in the medial part of the ventral horn. They were likely interneurons. In few instances putative motoneurons located in the lateral part of the ventral horn were also recorded. We did not investigate their identity further. Since all these neurons responded similarly to the different tests of our study, they were pooled in a single sample. We evoked pairs of monosynaptic EPSCs by stimulating a nearby region with a bipolar electrode (**Figures 2A,B**; see Materials and Methods). The second EPSC was bigger, similar or smaller than the first. Consequently, the paired pulse ratio (PPR), calculated as the relative value of the second and the first EPSCs was either below or above 1 (**Figure 1C**). To investigate if and how astrocytes modulate synaptic transmission, we used a second pipette filled with patch solution enriched with a high concentration of the Ca^2+^ chelator BAPTA (30 mM; see Materials and Methods). We approached it close to a neighboring fluorescent astrocyte (**Figures 1A,B**). Recording the astrocyte in cell-attached mode did not affect the evoked EPSCs (**Figure 2D**). However, few minutes (3–21) after breaking-in to whole-cell configuration, the amplitude of the first EPSC was significantly increased (**Figures 2B–D**; *n* = 6; significant increase for five of six cells considered individually; 6.8^*^10^-13^ < *p* < 0.033; two sample *t*-test and two sample Kolmogorov–Smirnov; significant increase for the mean values of all cells pooled together; 105 ± 142 pA to 135 ± 179 pA, *p* = 0.03; Wilcoxon signed-rank test). This result suggests that astrocytes exert a tonic inhibition on ventral horn neurons by releasing a gliotransmitter through a Ca^2+^ dependent mechanism. To determine if the inhibition occurred pre- or postsynaptically, we considered the response induced by the second stimulation. On average, chelating Ca^2+^ in the astrocyte had no significant effect on the second EPSC (**Figure 2E**; 95 ± 63 pA to 97 ± 68 pA; *n* = 6; *p* = 0.69; Wilcoxon signed-rank test). Consequently the PPR was decreased in all the cells tested (**Figures 2F–H**; significant decrease for all the cells pooled together; 1.4 ± 0.5 to 1.0 ± 0.3, *p* = 0.03 Wilcoxon signed-rank test). The input resistance of the recorded neurons was not changed after chelating Ca^2+^ in astrocytes (514 ± 298 MΩ in control; 507 ± 284 MΩ after Ca^2+^ chelating, *n* = 5; *p* = 0.3; Wilcoxon signed-rank test). Altogether these results suggest that astrocytes produce a tonic inhibition in the ventral horn by releasing a gliotransmitter that inhibits excitatory transmission at the presynaptic level.

### SPINAL ASTROCYTES GENERATE PHASIC PRESYNAPTIC INHIBITION

**FIGURE 3:**
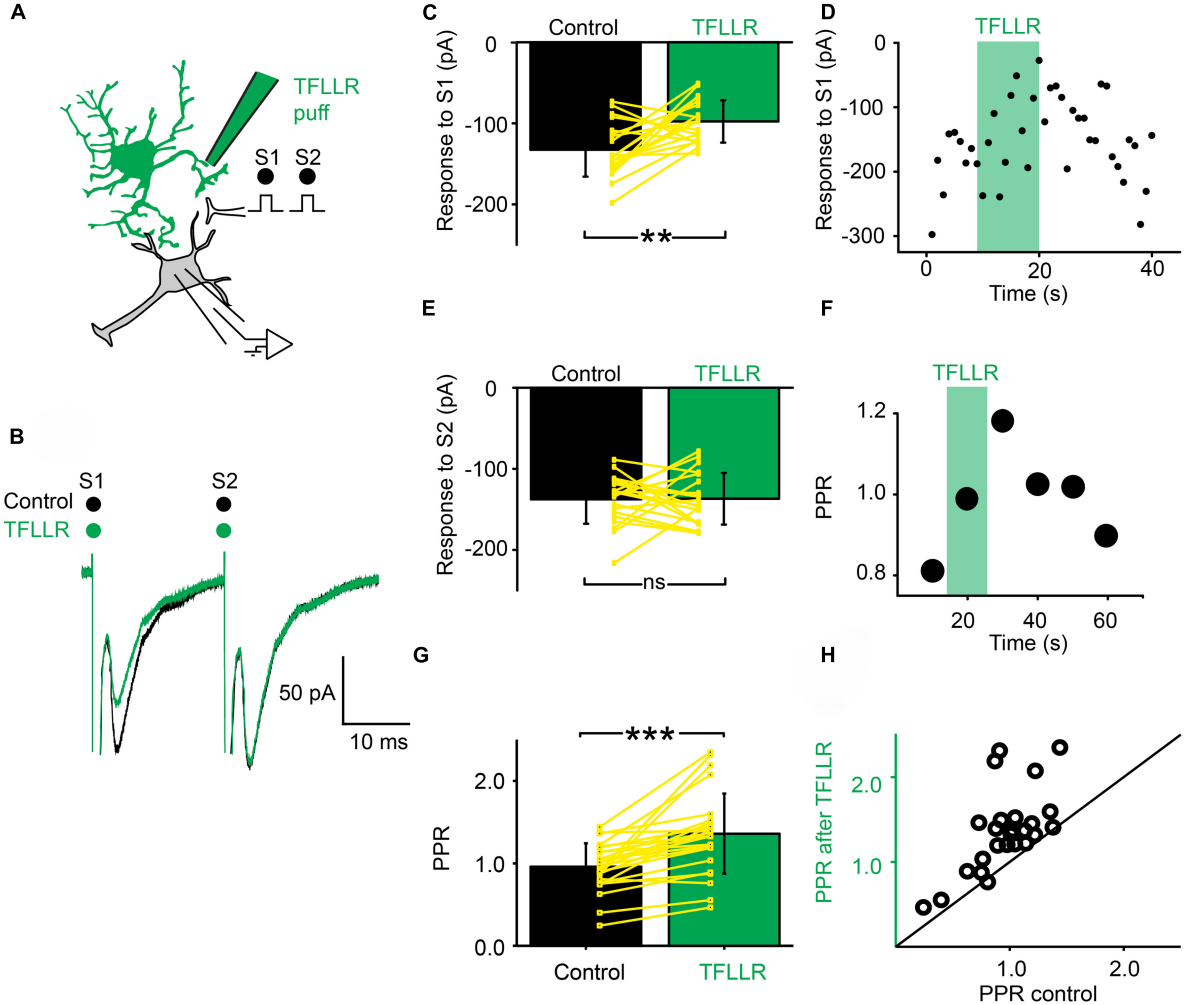
**Activation of astrocytes with TFLLR produces presynaptic inhibition of synaptic transmission. (A)** Schema of the principle of the preparation. Ventral horn neuron recorded in whole-cell mode. Synaptic stimulation produced by a bipolar electrode (S1, S2). Puff application of TFLLR. **(B)** Response of a ventral horn neuron to two stimulations in control conditions (black) and after puffing TFLLR between the stimulation and recording electrode (green). The amplitude of the first EPSC was decreased. **(C)** Mean amplitude of the first EPSC in control condition and after puffing TFLLR. The amplitude was significantly decreased. ^**^p < 0.01. **(D)** Amplitude of the first EPSC before, during (green bar) and after puffing TFLLR. Note the decrease in amplitude during the puff. **(E)** Amplitude of the second EPSC in control conditions and after puffing TFLLR. **(F)** Time course of the changes occurring for the PPR calculated for the neuron recorded in (B). TFLLR increased the PPR. **(G)** Mean PPR for all the cells recorded before and after puffing TFLLR. The yellow lines correspond to individual values. The PPR was significantly increased. ^***^p < 0.001. **(H)** PPR after TFLLR in one astrocyte as a function of PPR in control conditions. Most points are above the line of equality.

Next, we investigated if a phasic activation of astrocytes also produced an inhibition of synaptic transmission. For this purpose, we used a pipette filled with the peptide TFLLR, an agonist for the G-protein coupled PAR-1 receptor that is preferentially expressed in astrocytes (Lee et al., 2007; Shigetomi et al., 2008). TFLLR induces a Ca^2+^ increase in astrocytes (Lee et al., 2007; Shigetomi et al., 2008; Wang et al., 2013). When we puffed TFLLR between the stimulation and recording electrodes (**Figure 3A**), the amplitude of the first EPSC evoked by a paired stimulation protocol was significantly decreased (**Figures 3B–D**; *n* = 25; significant decrease for 20/25 cells tested individually; 3.3^*^10^-6^ < *p* < 0.05; paired sample *t*-test, Kolmorogov–Smirnov test and two sample *t*-test; significant decrease for all the cells pooled together; *p* = 1.9^*^10^-7^, paired sample *t*-test). The second EPSC was not significantly affected by TFLLR (**Figure 3E**; *n* = 25; *p* = 0.06; paired *t*-test). Consequently the PPR was increased (**Figures 3F–H**; *n* = 25; significant increase for all cells taken together; *p* = 1.7^*^10^-5^, paired *t*-test). The effect of TFLLR was correlated with the PPR observed under control conditions: the inhibition was stronger for synapses with a high release probability (PPR < 1; significant correlation; *p* = 0.04; *R* = -0.4; Pearson correlation test; *n* = 25). Since neither the input resistance of the postsynaptic neuron, nor the holding current were affected by TFLLR (control input resistance: 730 ± 531 MΩ; after TFLLR: 741 ± 572 MΩ, *n* = 23; *p* = 0.36, Wilcoxon signed-rank test; control holding current: -63 ± 69 pA; after TFLLR: -67 ± 75 pA, *n* = 25; *p* = 0.11, Wilcoxon signed-rank test), our results suggest that activation of astrocytes triggers the release of a substance that produces presynaptic inhibition of excitatory synaptic transmission in the ventral horn of the spinal cord. To identify the gliotransmitter involved, we performed a range of pharmacological tests.

### DUAL MODULATION OF SYNAPTIC TRANSMISSION BY ATP

**FIGURE 4:**
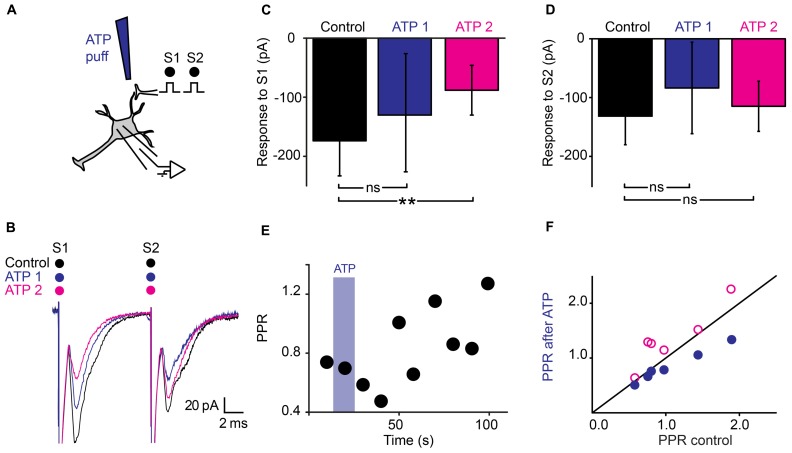
**Dual effects of ATP on synaptic transmission. (A)** Schema of the principle of the preparation. Ventral horn neuron recorded in whole-cell mode. Synaptic stimulation produced by a bipolar electrode (S1, S2). Puff application of ATP. **(B)** Response of a ventral horn neuron to two stimulations in control conditions (black), during (ATP 1) and 1 min after puffing ATP (ATP 2) between the stimulation and recording electrode. The amplitude of the first EPSC got more decreased with time while the amplitude of the second EPSC got less decreased. **(C)** Amplitude of the first EPSC in control condition, during a puff of ATP (ATP 1) and 1 min after (ATP 2). After 1 min, the amplitude was significantly decreased. ^**^p < 0.01. **(D)** Amplitude of the second EPSC in control conditions, during a puff of ATP (ATP 1) and 1 min after (ATP 2). The difference is not significant for this particular example **(E)** Time course of the changes in PPR induced by ATP for the cell recorded in (B). Note the initial decrease followed by an increase. **(F)** PPR calculated during (blue filled circles), and 1 min after puffing ATP (purple open circles) as a function of PPR in control conditions. After 1 min, most points are above the line of equality. Significant increase.

Since purines are major intrinsic modulators in the spinal cord (Dale and Gilday, 1996) that can be released by astrocytes (Pascual et al., 2005; Fields and Burnstock, 2006; Panatier et al., 2011), we tested the effect of ATP on synaptic transmission (**Figure 4A**). A 10 s Puff of ATP (1 mM) between the stimulation and recording electrodes reduced the amplitude of evoked pairs EPSCs (**Figure 4B**; *n* = 6). A careful inspection of the recordings revealed that the effects induced by ATP evolved with time. During the puff itself, the amplitude of the first EPSC was reduced of 18% (**Figure 4C**; from 131 ± 72 pA to 108 ± 37 pA; non-significant reduction, *p* = 0.22, Wilcoxon signed-rank test) while the second EPSC was decreased of 30% (**Figure 4D**; from 132 ± 71 pA to 93 ± 45 pA; significant reduction for all the cells tested together; *n* = 6; *p* = 0.03; Wilcoxon signed-rank test). Consequently, The PPR was significantly reduced (**Figures 4E,F**; from 1.08 ± 0.5 in control conditions to 0.85 ± 0.3 during ATP; *p* = 0.03, Wilcoxon signed-rank test). The effects initially induced by ATP were concomitant with a decrease in input resistance of postsynaptic neurons (from 480 ± 318 MΩ to 442 ± 295 MΩ; *p* = 0.03, Wilcoxon signed-rank test), suggesting a mixture of pre- and postsynaptic effects. However, 1 min after the puff, the effects induced by ATP were different. The response to the first chock was more decreased (**Figures 4B,C**; from 131 ± 72 pA to 81 ± 35 pA; *n* = 6; significant decrease for 4/6 cells, 0.0015 < *p* < 0.019, two sample *t*-test; significant decrease for all cells pooled together, *p* = 0.03, Wilcoxon signed-rank test), while the response to the second chock was less affected (**Figure 4D**; from 132 ± 71 pA to 109 ± 59 pA, significant decrease for all cells pooled together, *p* = 0.03, Wilcoxon signed-rank test). Thus, after an initial decrease, ATP induced an increase of the PPR (**Figures 4E,F**; from 1.08 ± 0.5 to 1.35 ± 0.5, *p* = 0.03, Wilcoxon signed-rank test). Since 1 min after the puff, the input resistance of the recorded neuron was not significantly different from control conditions (480 ± 317 MΩ in control; 500 ± 363 MΩ 1 min after ATP; *p* = 0.6, Wilcoxon signed-rank test), our results suggest that ATP induced presynaptic inhibition of transmitter release.

### ADENOSINE INDUCES PRESYNAPTIC INHIBITION

**FIGURE 5:**
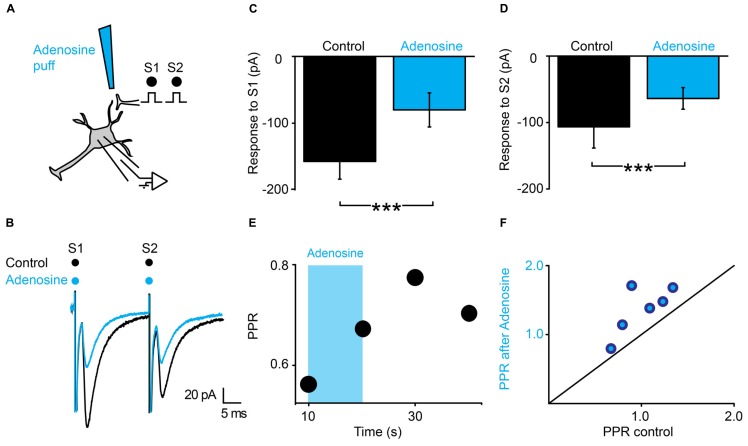
**Adenosine generates presynaptic inhibition. (A)** Schema of the principle of the preparation. Ventral horn neuron recorded in whole-cell mode. Synaptic stimulation produced by a bipolar electrode (S1, S2). Puff application of adenosine. **(B)** Response of a ventral horn neuron to two stimulations in control conditions (black) and after puffing adenosine between the stimulation and recording electrode (blue). The amplitude of both EPSCs was decreased. **(C)** Amplitude of the first EPSC in control condition and after puffing adenosine. Significant decrease. ^***^p < 0.001. **(D)** Amplitude of the second EPSC in control condition and after puffing adenosine. Significant decrease. ^***^p < 0.001. **(E)** Time course of the changes in PPR induced by adenosine (blue bar) for the cell recorded in (B). **(F)** PPR after puffing adenosine as a function of PPR in control conditions. All points are above the line of equality. Adenosine significantly increased the PPR.

The latency of the effects induced by ATP suggests that presynaptic inhibition is not induced by ATP itself, but by one of its metabolic products. Since ATP gets converted into adenosine by extracellular ectonucleotidases (Fields and Burnstock, 2006), we tested the effect of a puff of adenosine (1 mM), using the same protocol (**Figure 5A**). We found that adenosine induced a strong inhibition of the first EPSC (**Figure 5C**; *n* = 6; from 133 ± 134 pA to 64 ± 52 pA; significant decrease for 5/6 cells; 5.9^*^10^-17^ < *p* < 0.002; two sample *t*-test and two sample Kolmogorov–Smirnov; significant decrease for all cells pooled together, *p* = 0.03, Wilcoxon signed rank test) and a weaker inhibition of the second EPSC (**Figure 5D**; *n* = 6; from 141 ± 170 pA to 85 ± 76 pA; significant decrease for 4/6 cells; 1.2^*^10^-12^ < *p* < 0.008, two sample *t*-test and two sample Kolmogorov–Smirnov; non-significant decrease for all cells pooled together, *p* = 0.16, Wilcoxon signed rank test). Here also, the PPR was significantly increased (**Figure 5E**; from 1.0 ± 0.3 to 1.4 ± 0.4; *p* = 0.03, Wilcoxon signed-rank test). In contrast with ATP, the effects of adenosine were homogenous and occurred without significant change in input resistance (1007 ± 794 MΩ in control; 949 ± 680 MΩ after adenosine; *p* = 0.8; Wilcoxon signed-rank test). Altogether, these results suggest that adenosine inhibits EPSCs via a presynaptic mechanism.

### THE SELECTIVE BLOCKADE OF ECTONUCLEOTIDASES PREVENTS THE EFFECTS OF TFLLR

**FIGURE 6:**
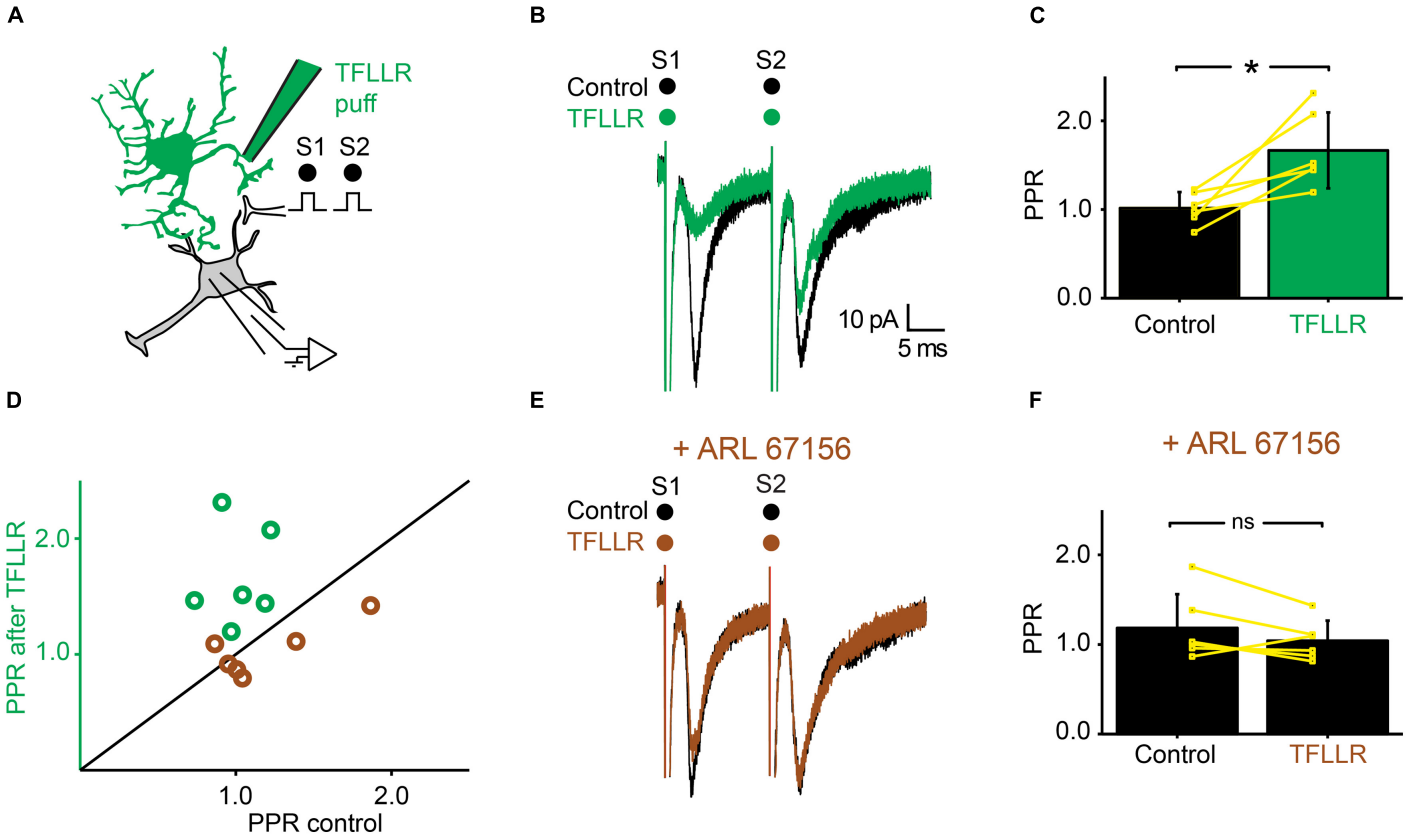
**Inhibiting extracellular ectonucleotidases blocks the effects of TFLLR. (A)** Schema of the principle of the preparation. Ventral horn neuron recorded in whole-cell mode. Synaptic stimulation produced by a bipolar electrode (S1, S2). Puff application of TFLLR. **(B)** Response of a ventral horn neuron to two stimulations in control conditions (black) and after puffing TFLLR between the stimulation and recording electrode (green). The amplitude of the first EPSC was decreased. **(C)** Mean values of the PPR before and after puffing TFLLR. ^*^p < 0.05. **(D)** PPR after puffing TFLLR as a function of PPR before puffing TFLLR. In control conditions, the PPR was significantly increased (green open circles). In the presence of ARL 67156, the PPR was not significantly increased anymore (brown open circles). **(E)** Same neuron as in **(B)**, recorded after addition of ARL 67156 to the bath. Puffing TFLLR did not affect the amplitude of the EPSC anymore (brown trace). **(F)** Mean values of the PPR before and after puffing TFLLR in the presence of ARL 67156.

We tested further the possible involvement of ATP in presynaptic inhibition by means of the very selective ectonucleotidase inhibitor ARL 67156 (Levesque et al., 2007). We selected neurons for which TFLLR induced presynaptic inhibition (**Figures 6A,B**). After addition of ARL 67156 (50 μM) to the extracellular medium, the first EPSC was not affected anymore by a puff of TFLLR (**Figure 6E**; *n* = 6; EPSC in normal Ringer: -76.2 ± 30 pA; after TFLLR: -52.0 ± 31.6 pA; significant decrease for 4/6 cells, 3.3^*^10^-6^ < *p* < 0.047, two sample *t*-test and two sample Kolmogorov–Smirnov; significant decrease for all cells pooled together, *p* = 0.03, Wilcoxon signed-rank test; EPSC in ARL 67156: 70.6 ± 44.3 pA; after TFLLR: 73.2 ± 48.9 pA; no significant decrease for cells considered individually 0.11 < *p* < 0.81, two sample *t*-test and Kolmogorov–Smirnov; no significant decrease for all cells pooled together, *p* = 0.7, Wilcoxon signed-rank test). Moreover, the PPR was not significantly affected (**Figures 6D,F**; *n* = 6: PPR in normal Ringer: 1.0 ± 0.2; TFLLR: 1.7 ± 0.4; *p* = 0.03 Wilcoxon signed-rank test; PPR in ARL 67156: 1.2 ± 0.4; TFLLR: 1.0 ± 0.2; *p* = 0.2 Wilcoxon signed-rank test). We therefore conclude that presynaptic inhibition is not mediated by ATP, but rather by one of its metabolic products.

### TFLLR INDUCES PRESYNAPTIC INHIBITION BY ACTIVATING ADENOSINE A_1_ RECEPTORS

**FIGURE 7:**
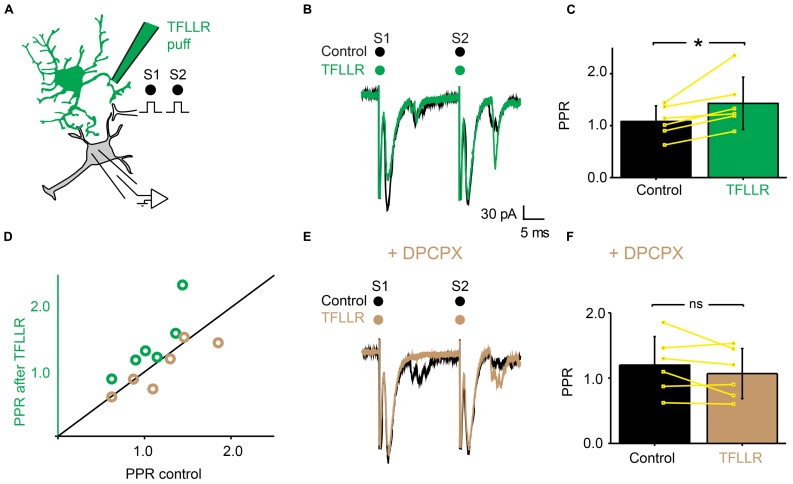
**The effects of TFLLR are blocked by DPCPX. (A)** Schema of the principle of the preparation. Ventral horn neuron recorded in whole-cell mode. Synaptic stimulation produced by a bipolar electrode (S1, S2). Puff application of TFLLR. **(B)** Response of a ventral horn neuron to two stimulations in control conditions (black) and after puffing TFLLR between the stimulation and recording electrode (green). The amplitude of the first EPSC was decreased. **(C)** Mean values of the PPR before and after puffing TFLLR. ^*^p < 0.05. **(D)** PPR after puffing TFLLR as a function of PPR before puffing TFLLR. In control conditions, TFLLR induced an increase of the PPR (green circles). In the presence of DPCPX, TFLLR did not change the PPR (beige circles). **(E)** Same neuron as in (B), recorded after addition of DPCPX to the bath. Puffing TFLLR did not affect the amplitude of the EPSP anymore (beige trace). **(F)** Mean values of the PPR before and after puffing TFLLR in the presence of DPCPX.

Since adenosine induced presynaptic inhibition (**Figure 5**), we tested if blocking adenosine A_1_ receptors prevented the induction of presynaptic inhibition by TFLLR (**Figure 7A**). We found that in the presence of the selective adenosine A_1_ receptor antagonist DPCPX (5 μM), puffing TFLLR did not affect the amplitude of evoked EPSCs anymore (**Figures 7B,E**; *n* = 6; effect of TFLLR in control conditions: from 153 ± 63 pA to 111 ± 47; significant decrease for 6/6 cells, 0.0017 < *p* < 0.043, two sample *t*-test and two sample Kolmogorov–Smirnov; significant decrease for all cells pooled together, *p* = 0.03, Wilcoxon signed-rank test; effect of TFLLR in DPCPX: from 135 ± 63 pA to 131 ± 58 pA; no significant decrease for cells considered individually: 0.18 < *p* < 0.71, two sample *t*-test and Kolmogorov–Smirnov; no significant decrease for all cells pooled together; *p* = 0.3, Wilcoxon signed-rank test). The PPR, which was significantly increased in control conditions (**Figures 7C,D**), was not altered anymore (**Figures 7D,F**; *n* = 6; increase of PPR in control conditions: from 1.1 ± 0.3 to 1.4 ± 0.5; *p* = 0.03, Wilcoxon signed-rank test; no change of PPR in DPCPX: from 1.2 ± 0.4 to 1.1 ± 0.4; *p* = 0.3, Wilcoxon signed-rank test). Our results demonstrate that the metabolic product of ATP that induces presynaptic inhibition of excitatory synaptic transmission is likely to be adenosine acting on A_1_ receptors.

## DISCUSSION

**FIGURE 8:**
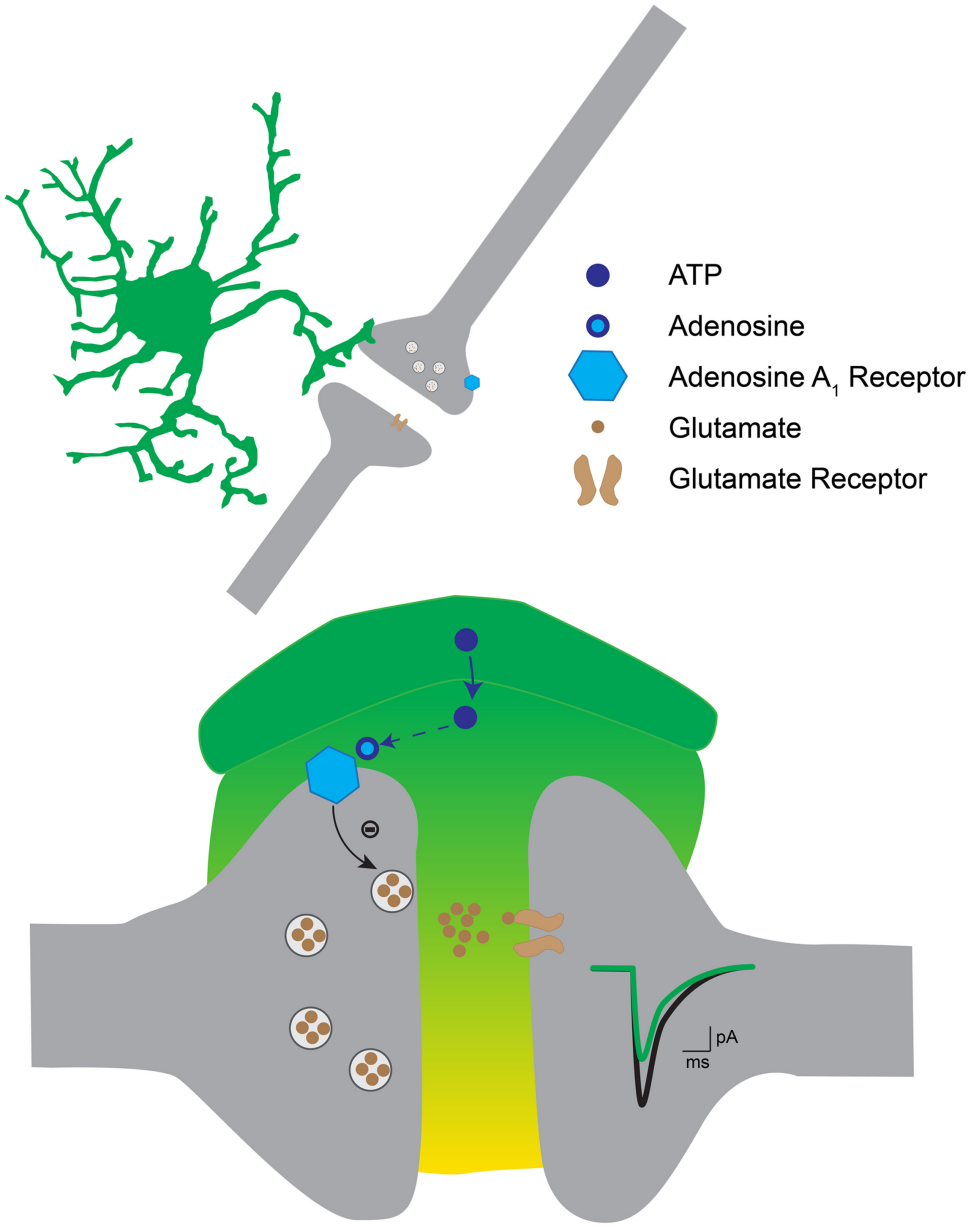
**Hypothetical mechanism for astrocytic induced presynaptic inhibition in the ventral horn.** The activation of astrocytes induces the release of ATP, which gets converted into adenosine by extracellular ectonucleotidases. Adenosine binds to presynaptic A_1_ receptors on excitatory terminals. This in turn decreases the probability of transmitter release. As a result excitatory postsynaptic currents are decreased.

Our study suggests that astrocytes located in the ventral horn of the spinal cord induce both tonic and phasic presynaptic inhibition of excitatory synaptic transmission. Our results indicate that extracellular ectonucleotidases convert ATP released by astrocytes into adenosine, which binds to A_1_ inhibitory receptors located on the presynaptic side of excitatory synapses (**Figure 8**).

### THE MODULATION INDUCED BY ASTROCYTES IS BOTH TONIC AND PHASIC

Chelation of Ca^2+^ in one astrocyte induced a decrease of the PPR suggesting a removal of presynaptic inhibition (**Figure 2**). The most likely mechanism is an increase of the residual Ca^2+^ present in presynaptic terminals since both the release probability (*p*) and the pool of available quanta (*n*) are Ca^2+^ dependent (Zucker and Regehr, 2002). Even though the conductance measured in postsynaptic neurons was unaffected by the BAPTA treatment, we cannot rule out postsynaptic mechanisms (for example if there are two or more conductances affected in opposite ways).

This result suggests that astrocytes continuously release purines that produce presynaptic inhibition that might contribute to lower the activity of the spinal motor network. Tonic release of vesicular ATP from astrocytes was previously reported in the hippocampus (Pascual et al., 2005). Released ATP gets converted into adenosine by extracellular ectonucleotidases. This mechanism explains the persistent synaptic suppression mediated by adenosine (Pascual et al., 2005). The central nervous system uses different strategies to decrease its excitability, the most common being a general increase of membrane conductance provided by an ambient concentration of GABA (Farrant and Nusser, 2005; Bautista et al., 2010).

Puffing TFLLR produced a transient inhibition of excitatory synaptic transmission (**Figure 3**). This suggests that the level of presynaptic inhibition provided by astrocytes is not only tonic but can also be regulated by the activity of the network. TFLLR is an exogenous molecule and does not activate astrocytes under physiological conditions. We did not investigate the nature of endogenous neurotransmitters responsible for the activation of astrocytes. The most likely scenario is that glutamate released from excitatory synapses binds to receptors located in the membrane of neighboring astrocytes that would in turn release ATP. Such a mechanism has been described in slices from the hippocampus where glutamate binding to metabotropic glutamate subtype 5 receptors triggers the release of ATP from astrocytes (Zhang et al., 2003; Panatier et al., 2011). Another possibility could be a change in pH. Brain hypoxia and ischemia lead to acidosis (Tombaugh and Sapolsky, 1993), which induces the release of ATP from astrocytes in the brainstem (Gourine et al., 2010). This process is essential for adjusting respiratory responses. These possibilities will be tested during future experiments.

The interpretation of our results relies on the hypothesis that the number of axons recruited by the electrical stimulation remained constant during each experiment. Puffing drug with pressure could in principle induce a mechanical artifact resulting in a change of the excitability of stimulated fibers. However, if it was the case, the number of recruited axons would either increase or decrease in a random manner. In contrast, puffing TFLLR induced either no effect or a reliable decrease of synaptic transmission. In addition, when blocking ectonucleotidases or A_1_ receptors abolished the effect of TFLLR puff (see **Figures 6** and **7**), which rules out the possibility of a substantial change in the number of recruited axons.

### IDENTITY OF THE PURINES AND OF THE RECEPTORS RESPONSIBLE FOR PRESYNAPTIC INHIBITION

Our results demonstrate that presynaptic inhibition is triggered by ATP, which, after enzymatic hydrolysis to adenosine binds to presynaptic receptors. Several arguments support this interpretation. First, presynaptic inhibition induced by TFLLR was both reproduced by ATP and adenosine (**Figures 4** and **5**). However, in contrast to adenosine, the inhibition produced by ATP was complex since, during the puff application, it was characterized by a decrease in PPR, which is not compatible with presynaptic inhibition. After 1 min, the inhibition produced by ATP was characterized by an increase in PPR, like the effect induced by adenosine. This suggests that adenosine rather than ATP is responsible for presynaptic inhibition. This interpretation is supported by the fact that the conversion of ATP to adenosine occurs within a range of 200 ms (Dunwiddie et al., 1997). Second, blocking the hydrolysis of ATP to adenosine by inhibiting extracellular ectonucleotidases with ARL 67156 suppressed the presynaptic inhibition triggered by TFLLR (**Figure 6**). This confirms that ATP itself does not induce presynaptic inhibition and suggests instead that a metabolic product of ATP such as ADP, AMP, or adenosine is involved (Fields and Burnstock, 2006). Third, blocking adenosine A_1_ receptors with DPCPX suppressed the effects of TFLLR (**Figure 7**). This demonstrates that the purine responsible for presynaptic inhibition is actually adenosine.

Adenosine inhibits synaptic release of transmitter in other systems such as the dentate gyrus (Dolphin and Archer, 1983) and the CA1 region of the hippocampus (Corradetti et al., 1984; Wu and Saggau, 1994), the basal forebrain (Hawryluk et al., 2012), and the cerebral cortex (Hollins and Stone, 1980). In most cases, presynaptic adenosine A_1_ receptors decrease transmitter release by inhibiting a Ca^2+^ current (Hollins and Stone, 1980; Wu and Saggau, 1994; Dale and Gilday, 1996; Brown and Dale, 2000; Zhang et al., 2003; Fields and Burnstock, 2006). In contrast, adenosine A_2A_ receptors facilitate synaptic transmission (Panatier et al., 2011). In agreement, we found that A_1_ receptors mediate presynaptic inhibition in the ventral horn of the spinal cord (**Figure 7**).

### FUNCTIONAL RELEVANCE

Regulating the excitability motor networks is essential for adjusting the motor command. Hyperexcitability of motor networks leads to involuntary movements and spasms. Tonic presynaptic inhibition of excitatory transmission may provide a general decrease of the activity of highly active networks and act as a homeostatic mechanism. In other regions of the central nervous system such as the hippocampus, adenosine released from astrocytes has neuroprotective effects and the activation of presynaptic A_1_ receptors acts as en endogenous anticonvulsant (Boison, 2012). In the spinal cord, artificial spinal rhythmic activities induced by cocktails of pharmacological agents are modulated by adenosine. The activation of A_1_ receptors depresses bicuculline-evoked seizures in newborn rats (Brockhaus and Ballanyi, 2000; Taccola et al., 2012). This suggests that adenosine also has anticonvulsant effects in the spinal cord. Indeed, in the spinal cord of young rats, acute hypoxia triggers the release of adenosine, which in turn depresses monosynaptic reflexes (Otsuguro et al., 2011). Such a neuroprotective role of adenosine is well documented in patients suffering from temporal lobe epilepsy. Seizures trigger a massive release of adenosine, which in turn activate A_1_ receptors and thereby terminate the activity (Boison, 2012).

Adenosine might also be involved in the tuning of locomotor activity. Antagonists for adenosine receptors from the Methylxanthine family (such as caffeine or theophylline) increase locomotor activity (Snyder et al., 1981). During fictive locomotion induced in neonatal mice by a mixture of serotonin, NMDA and dopamine, bath application of adenosine slows down rhythmic activity by around 30% (Witts et al., 2012). To figure out the actual physiological role of purines during locomotion in adult mammals, one will have to measure their concentrations. The recent development of probes allowing the detection of ATP and adenosine in real time (Frenguelli et al., 2007) may allow figuring out if the concentration of purines changes tonically or rhythmically during movement. Interestingly, ATP increases the excitability of spinal locomotor networks (Dale and Gilday, 1996). Since the hydrolysis of ATP to adenosine occurs one range of magnitude faster than the locomotor cycle (Dunwiddie et al., 1997), a possible scenario could be that astrocytes release ATP cyclically, in phase with the rhythm. The fast degradation of ATP into adenosine would then induce a rhythmic inhibition of excitatory transmission. However, high-speed locomotion could be too fast for rhythmic mediated inhibition. We will test these hypotheses during future experiments.

## Conflict of Interest Statement

The authors declare that the research was conducted in the absence of any commercial or financial relationships that could be construed as a potential conflict of interest.
